# Omics-Level Approaches to Studying Gammaherpesvirus Infection

**DOI:** 10.3390/pathogens15070713

**Published:** 2026-07-07

**Authors:** Fatima Hisam, Anisha Reddy Konakalla, Eranda Berisha, Maria del Carmen Chacon Castro, Spandan Mukherjee, Claire Wang, Benjamin R. Sheirbon, Tracie Delgado, Erica L. Sanchez

**Affiliations:** 1 Department of Biological Sciences, The University of Texas at Dallas, Richardson, TX 75080, USAanishareddy.konakalla@utdallas.edu (A.R.K.); eranda.berisha@utdallas.edu (E.B.); mariadelcarmen.chaconcastro@utdallas.edu (M.d.C.C.C.); spandan.mukherjee@utdallas.edu (S.M.); claire.wang@utdallas.edu (C.W.); 2 Department of Biology, Seattle Pacific University, Seattle, WA 98119, USA; sheirbonb@spu.edu (B.R.S.); delgadot@spu.edu (T.D.)

**Keywords:** genomics, transcriptomics, proteomics, metabolomics, KSHV, EBV, MHV-68, multi-omics, gammaherpesvirus

## Abstract

Gammaherpesviruses (GHVs) represent a global clinical burden as the causative agents of Kaposi’s sarcoma and mononucleosis, among other diseases. Kaposi’s sarcoma-associated herpesvirus (KSHV) and Epstein–Barr virus (EBV) are the most studied human GHVs, and murine gammaherpesvirus 68 (MHV-68) is a recognized experimental model. GHVs are defined by their modulation of the host cell to establish lifelong latent infections and increase host dysregulation during periodic reactivation. Due to their ubiquitous changes in host cells, systems-level techniques are well-suited to study GHV infections at all stages of the central dogma: genomics, transcriptomics, and proteomics. Furthermore, metabolomics can reveal the final metabolic changes across numerous host cellular pathways. This review assesses the current knowledge on GHV infections gained through omics techniques. We also identify gaps and propose future directions, including the development of new therapeutic strategies. Early omics techniques have characterized large swaths of infection for EBV, KSHV, and MHV-68, revealing conserved genes, homologous transcripts, and proteins. Modern omics techniques have enabled higher-resolution studies, yielding insights into heterogeneity in viral-host gene, transcript, and protein modulation strategies across geographical populations, viral subtypes, inter- and intra-patient infections, and latent and lytic states. The metabolome during GHV infections remains the least understood, but current studies have identified essential modulations of nucleotide, amino acid, and lipid synthesis by EBV, KSHV, and MHV-68. Importantly, the application of integrative omics methods to GHV infections remains a promising direction of study as the increased resolution of modern techniques meets the need for greater understanding of differences in each GHV infection.

## 1. Introduction

Gammaherpesviruses (GHVs) are a biologically and clinically relevant subfamily of *Herpesviridae*, defined by their capacity to establish lifelong latent infections in host cells, with potential for periodic reactivation [1]. These viruses have co-evolved with their hosts to exploit cellular processes while evading immune surveillance. Among the GHVs, Epstein–Barr virus (EBV) and Kaposi’s sarcoma-associated herpesvirus (KSHV) are the most well-characterized human pathogens, whereas murine gammaherpesvirus 68 (MHV-68) serves as a widely used experimental model for studying GHV pathogenesis in vitro and in vivo [2].

EBV, the first human oncogenic virus to be discovered, infects over 90% of the adult population worldwide and is associated with a broad spectrum of lymphoproliferative disorders and malignancies, including Burkitt’s lymphoma (BL), Hodgkin’s lymphoma (HL), nasopharyngeal carcinoma (NPC), and various post-transplant lymphoproliferative diseases [3,4,5,6]. KSHV, while less ubiquitous, is the etiologic agent of Kaposi’s Sarcoma (KS), Primary Effusion Lymphoma (PEL), Multicentric Castleman Disease (MCD), and Kaposi’s Sarcoma Inflammatory Cytokine Syndrome (KICS) [7]. Diseases arising from KSHV infection predominantly impact immunocompromised individuals [8]. MHV-68 does not naturally infect humans; however, the virus shares substantial genomic and functional homology with EBV and KSHV and recapitulates many key features of GHV infections in mice, including latency in B cells, tumor formation, lytic reactivation, and immune modulation [9]. Overall, these three GHVs collectively provide a framework for understanding the fundamental biology and disease mechanisms during oncogenic herpesvirus infections. Viruses of the sub-family *Gammaherpesvirinae* are found in New World and Old World primates, with only EBV and KSHV known to cause pathogenesis in humans [10].

The lifecycle of GHVs is characterized by two distinct phases: latency, during which the viral genome persists as an episome with limited gene expression and no virion production; and lytic replication, which involves the coordinated expression of all viral genes, viral genome replication, and the production of infectious virions [1]. Transitions between latency and lytic replication are regulated by a complex network of viral and host factors and are influenced by environmental and immunological cues. This dynamic nature between long-term latency and periodic acute lytic replication not only influences viral persistence and transmission but also the pathogenesis of GHV-associated diseases [11,12,13].

**Figure 1:**
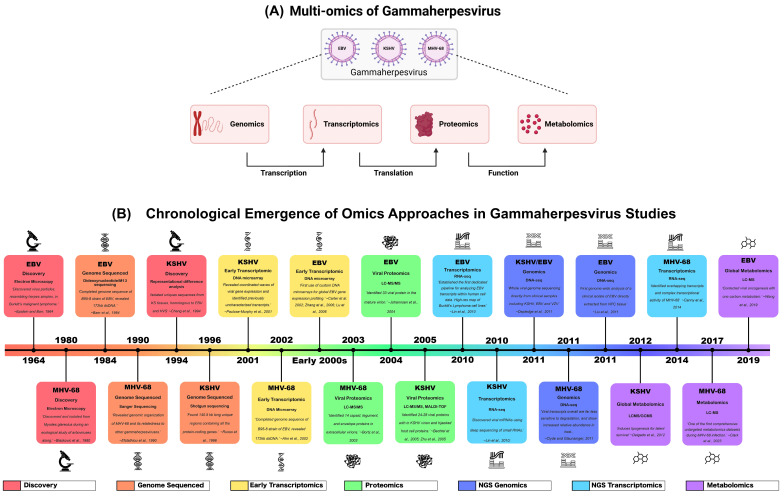
Multi-omics approaches to study gammaherpesvirus biology (**A**) Schematic overview of integrated omics strategies applied to gammaherpesviruses, including EBV, KSHV, and MHV-68. These approaches provide a systems-level view of viral gene expression, translation, and functional outcomes during infection. Created in BioRender. Lab, S. (2026) https://BioRender.com/571oiin (accessed on 8 June 2026). (**B**) Timeline for the introduction and progression of major omics technologies applied to the study of EBV [24,25,26,27,28,29,30,31,32], KSHV [16,33,34,35,36,37,38,39], and MHV-68 [2,40,41,42,43,44,45]. Key milestones include the discovery of the virus and genomics, transcriptomics, proteomics, and metabolomics approaches that advanced the understanding of viral biology, host–virus interactions, latency, lytic replication, and pathogenesis. Representative landmark studies implemented in gammaherpesvirus research are graphed chronologically. Image created in Adobe 2025, *Adobe Illustrator* (Version 29.8.7).

The emergence of high-throughput systems-level ‘omics’ technologies has transformed our ability to study host–virus interactions in a more comprehensive and unbiased manner [14]. Genomic approaches enable detailed analyses of viral genome structure, sequence diversity, and epigenetic modifications [15,16]. Transcriptomic profiling, particularly using next-generation RNA sequencing (RNA-seq), provides insights into viral and host gene expression dynamics during different stages of infection and across heterogeneous cell types and tissues [17]. Proteomics has defined the structural viral and functional viral and host proteome by revealing post-translational modifications (PTM), protein–protein interactions (PPI), and the assembly of multiprotein complexes that mediate key steps in the viral life cycle [18,19]. Finally, metabolomics through mass spectrometry (MS) and nuclear magnetic resonance (NMR) spectroscopy captures the metabolic rewiring induced by infection, shedding light on how GHVs manipulate host bioenergetics, nutrient sensing, and signaling pathways to favor their replication and persistence [20,21]. Together, these multi-omics approaches provide a systems-level view of GHV biology, allowing for the identification of critical nodes of viral control and host response [22] (Figure 1A). Moreover, the chronological emergence of omics technologies for the comparisons between EBV, KSHV, and MHV-68 has illuminated both conserved and divergent mechanisms employed by GHVs to establish persistent infections and evade immune detection [23] (Figure 1B).

In this review, we integrate current knowledge derived from genomics, transcriptomics, proteomics, and metabolomics studies of EBV, KSHV, and MHV-68 (Figure 1). We highlight how these approaches reveal the complex molecular modulation of GHV infections, identify key gaps in the field, and propose future research directions to inform diagnostic, therapeutic, and preventive strategies.

## 2. Genomics

**Figure 2:**
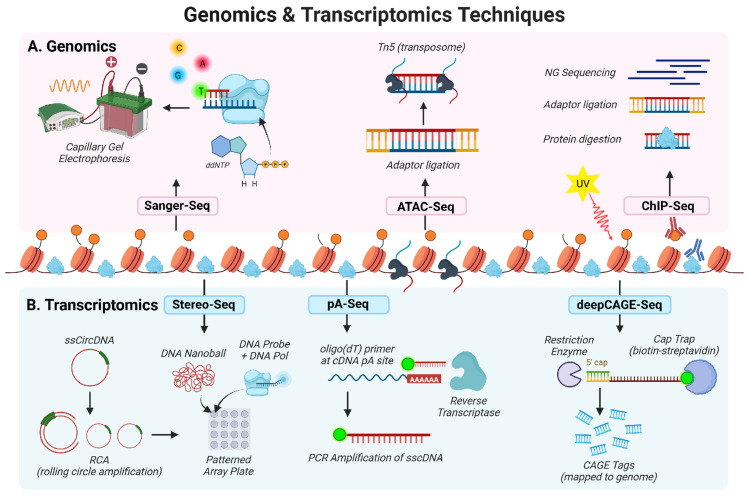
Genomics and transcriptomics techniques (left to right) (**A**) Genomics: Sanger sequencing, assays for transposase-accessible chromatin sequencing (ATAC-Seq), and chromatin immunoprecipitation sequencing (ChIP-Seq) were performed on uncoiled DNA, transposome-isolated DNA fragments, and antibody-bound histones and non-histones, respectively. (**B**) Transcriptomics. Stereo sequencing, polyadenylation sequencing (pA-Seq), and deep cap analysis of gene expression (deepCAGE-Seq) are all techniques performed on DNA or cDNA fragments. Created in BioRender. Lab, S. (2026) https://BioRender.com/l9f2o2d (accessed on 8 June 2026).

In virology, moving from studying individual genes to the study of entire genomes, or “genomics,” enabled the mapping of complete viral genomes, allowing researchers to identify all viral genes, regulatory elements, and their interaction with host DNA [46]. Early methods of Sanger sequencing paved the way for sequencing entire viral genomes, determining genetic organization, while high-throughput sequencing techniques, such as next-generation sequencing (NGS) and single-molecule real-time sequencing (SMRT), have revolutionized virology by allowing us to decode the full genome of a virus, identify mutations, and characterize viral diversity within and between hosts [47,48,49,50,51,52]. Genomic technologies have been essential in tracking viral outbreaks, monitoring drug resistance, and discovering novel viruses [53,54,55]. Recent innovations, long-read sequencing (LRS) and single-cell sequencing (sc-Seq), coupled with CRISPR/Cas-based approaches, have enhanced the resolution of genome studies, allowing for more accurate assembly of complex genomes in GHVs and providing insights into gene expression at the single-cell level [56,57,58]. Viral genomics has mapped the genomes of GHVs, which share large, linear double-stranded DNA genomes of 110–180 kb, encoding approximately 80–100 open reading frames (ORFs) [25,59,60,61]. Comparative genomics reveals high conservation in genes involved in viral replication: DNA polymerase BALF5 in EBV, ORF9 in KSHV and MHV-68; latency maintenance: EBNA1 in EBV, Latency-Associated Nuclear Antigen (LANA) in KSHV, and mLANA in MHV-68; and immune modulation: vIL-6 in KSHV and MHV-68. LANA in KSHV and mLANA in MHV-68 both tether the viral episome to host chromatin, ensuring genome persistence during cell division, which is a function analogous to EBNA1 in EBV [62,63,64]. Apoptotic inhibitors, vBCL-2 genes, are conserved across all three viruses (ORF16 in KSHV, BRF1 in EBV, and M11 in MHV-68), which underscores a shared strategy to evade host cell death and promote latency [65,66,67]. MHV-68 shares homologous ORFs with KSHV, like complement regulatory protein (ORF4), a D-type cyclin (ORF72), a viral G-protein-coupled receptor (ORF74), and an immune evasion protein (ORF K3) [61]. Comparative genomic studies have revealed that despite shared core genes and genome architecture, virus-specific adaptations such as KSHV-unique lytic gene cascade or EBV capacity for B-cell transformation contribute to their divergent pathogenic profiles [13,68,69] (Figure 2).

### 2.1. Genomic Approaches to Study Gammaherpesviruses

The genomic study of GHVs followed the initial discovery of EBV in 1964 using electron microscopy to visualize its presence in lymphoblasts isolated from Burkitt’s lymphoma tumors [24,70]. In 1984, the complete genome from the B95.8 type I EBV strain was fully sequenced using dideoxynucleotide/M13 sequencing, revealing its 172 kb linear dsDNA structure and identifying essential RNA polymerase II promoters and protein-coding regions, including a ribonucleotide reductase, a DNA polymerase, and two surface glycoproteins [25]. Later, Sanger sequencing of the Raji strain of EBV revealed the DNA sequence corresponding to the 12 kb region deleted in the B95.8 strain, identifying the three ORFs missing in the B95.8 strain and emphasizing the genomic variability within EBV [71,72].

MHV-68 was first discovered in 1980 following the isolation of herpesvirus strains from wild rodents, with electron microscopy confirming their classification within the *Herpesviridae* [40]. Sequencing of MHV-68 started in 1990 using Sanger sequencing, which revealed its genomic organization and demonstrated its relatedness to other herpesvirus subfamilies, particularly gammaherpesviruses such as EBV [2]. Cycle sequencing, an improved version of Sanger sequencing, led to the complete MHV-68 genome characterization, identifying 80 viral genes and their collinearity with GHVs (KSHV and EBV), including herpesvirus saimiri (HVS), an oncogenic virus of primates [61,73]. These studies established MHV-68 as a model for understanding GHV biology both in vitro and in vivo.

KSHV was identified in 1994 using representational difference analysis coupled with PCR, which enabled the identification of unique viral DNA fragments in KS tissues from AIDS patients [33]. Early sequence analysis of KSHV revealed homology to capsid and tegument genes of other gammaherpesviruses, particularly EBV and HVS, highlighting the close evolutionary relationship between KSHV and EBV within the *Gammaherpesvirinae* subfamily. This work led to the classification of the virus as the eighth human herpesvirus, HHV-8, now known as KSHV, with the detection of these viral sequences in over 90% of KS lesions, emphasizing the strong association of KSHV with KS.

Genomic characterization of KSHV was initiated in 1996 using shotgun sequencing and automated dideoxy cycle sequencing of cosmid and lambda phage libraries derived from the lymphoma BC-1 cell line [16]. This analysis revealed a unique 140.5 kb region flanked by terminal repeat sequences rich in G + C content and identified 81 ORFs encoding homologs to human cytokines, cell cycle regulators, and apoptosis inhibitors, demonstrating the virus’s ability to manipulate host pathways. In 1997, the shotgun sequencing of a 17 kb KSHV lambda clone obtained from a KS lesion identified a viral cyclin (v-cyclin) with homology to human cyclin D2 and HVS v-cyclin [74]. KSHV v-cyclin is associated with cellular CDK6 and induces its kinase activation, regulating the host cell cycle [74]. Moreover, shotgun sequencing of a 17 kb genomic fragment identified vIL-6, a KSHV-encoded homolog of interleukin-6, mirroring the role of human IL-6 in immune modulation and cell proliferation during KSHV pathogenesis [75].

Sanger whole-genome sequencing of EBV has provided critical insights into the genomic diversity, particularly within latency-associated genes, EBNA2, EBNA3A, EBNA3B, and EBNA3C, that define type I and type II viruses [76]. While core genomic regions remain conserved, certain genes, like LMP1, exhibit high variability, suggesting potential functional differences in viral signaling and pathogenesis. In addition, comparative analyses have identified non-conserved coding regions and novel regulatory features, including RNA editing events, highlighting the complexity and functional diversity of the EBV genome. High-throughput sequencing of EBV genomes from infected B cells revealed phylogenetic clustering and genomic variation linked to the geographic origin of the disease, which highlighted the viral diversity of EBV influencing pathogenesis and disease outcomes [77]. Another whole-genome sequencing study on EBV-infected B cells investigated virus-induced host genomic alterations and revealed copy number variations (CNVs) while identifying MUC19 as a key regulator of EBV latency, linking EBV-driven CNVs to viral latency and oncogenesis [78].

Next-generation sequencing (NGS) of geographically different EBV strains from diverse clinical and geographical sources, including cell lines, primary tumors, and blood samples from spontaneous lymphoblastoid cells, BL, HL, NPC, and gastric cancer (GC) cells, revealed that while overall genome organization is largely conserved, variation, including novel deletions and increased SNP density, is enriched in latency-associated genes, suggesting that EBV genetic diversity may influence disease context and host interactions [79]. Sequencing of EBV genomes from oral wash and B cell fractions of acute infectious mononucleosis (IM) patients through target enrichment (using biotinylated RNA probes) and deep sequencing revealed intra- and interpatient EBV genomic diversity, especially in latency-associated genes and structural genes such as envelope glycoproteins [80]. Target capture-based whole-genome sequencing analyses of EBV across diverse disease subjects and 8 different cell lines reveal extensive single-nucleotide variation and structural diversity in the viral genome, reinforcing the role of viral genomic heterogeneity in disease-specific pathogenesis [81]. Whole-exome sequencing revealed that pulmonary lymphoepithelial carcinoma has a low frequency of canonical lung cancer driver mutations (EGFR, KRAS, BRAF) but displays extensive copy number deletions that overlap with EBV integration sites, suggesting that EBV-driven host genomic instability and somatic copy number variant loss may contribute to tumorigenesis [82]. Whole-genome sequencing of KSHV genomes from Zambian KS biopsies using target enrichment and NGS revealed conserved central genomic regions and variability in the terminal regions [83]. This variability resulted in distinct phylogenetic clustering, suggesting geographic subtypes. Polymorphic regions, including K1, K15, and K4.2, were identified as hotspots for genetic diversity, highlighting their potential roles in immunomodulation and viral phenotypic diversity. Whole-genome sequencing studies, including short-read sequencing, long-read sequencing, and NGS of KS skin lesions, plasma, whole blood, and oral fluid samples across diverse populations, reveal extensive genomic diversity, including novel polymorphisms, recombination events, geographic clustering, and mixed infections within individuals, highlighting both global and intra-host variability [84,85,86].

Genomic approaches have also been extensively employed to characterize viral genomes and identify markers or risk variants associated with herpesvirus-related diseases. For example, samples from EBV-related tumors, saliva, and plasma were selected, and large-scale genome sequencing and a two-stage genome-wide association study (GWAS) were performed on EBV isolates from China [87]. Two non-synonymous EBV variants within the lytic gene, BALF2, were strongly associated with NPC risk. Phylogenetic analysis of the risk variants revealed a unique Asian origin and suggested a clonal expansion in NPC-endemic regions. Later, the EBV genome was sequenced from Japanese patients with NPC using the high-throughput NGS technique and compared to other EBV-associated diseases from Japan and NPC from Southern China [88]. BALF2 variations were observed in Japanese and southern Chinese NPCs, and specific risk variants were associated with each population. Population-scale genomic analyses demonstrate that viral persistence is influenced by both viral genetic variation and host genetic factors, highlighting the importance of host–virus genomic interactions in infection dynamics [89]. In parallel, functional genomic studies have identified high-risk viral variants associated with NPC that promote immune evasion, directly linking viral genotype to oncogenic potential [90]. In the case of KSHV, Illumina sequencing of DNA from a KSHV-positive patient’s blood sample enabled the first identification of co-infections with KSHV and HHV6a (betaherpesvirus), which recognized KICS as a distinct clinical condition [91]. Later, a PCR-based enrichment (AmpliSeq) and Ion Torrent sequencing analysis of the KSHV genome from a KICS patient tracked viral genetic changes over time, identifying markers like IL-6 and IL-10 as diagnostic tools to differentiate KICS from KS [92]. These findings highlight how geographic and genetic variability influence herpesvirus-associated disease development and demonstrate the critical role of genomic tools in advancing our understanding of these complex viral infections.

### 2.2. Epigenetic Modulation

Epigenetic modulation is a key mechanism by which DNA viruses regulate their gene expression to establish and maintain long-term latent infections within host cells [93]. Upon nuclear entry, viral genomes are chromatinized to host histone proteins and are regulated via covalent modifications such as methylation and acetylation. These modifications are deposited and removed by host epigenetic regulators, including histone methyltransferases like EZH2, histone acetyltransferases, and polycomb group proteins, which respectively mark viral chromatin with repressive modifications such as H3K27me3 or activating marks like H3K4me3 and histone acetylation. The interplay of histone marks controls the transition between heterochromatinized latent states and euchromatic active states required for viral replication [94,95,96]. The role of epigenetics in regulating EBV, MHV-68, and KSHV latency and reactivation has been progressively uncovered through multiple studies [15,97,98,99]. During KSHV latency, the viral genome has bivalently chromatinized domains at key immediate-early promoters that simultaneously carry activation and repression of H3K4me3 marks, enabling the virus to maintain a transcriptionally poised but repressed state [95]. Reactivation stimuli promote the removal of repressive marks and the recruitment of the viral replication and transcription activator (RTA), facilitating chromatin remodeling and the initiation of the lytic gene expression cascade [94,95,96]. Host innate immune factors, such as IFI16, can epigenetically repress EBV and KSHV lytic genes by binding viral promoters and recruiting chromatin modifiers to maintain latency [100,101]. Degradation of these factors alleviates repression to permit reactivation.

Assays for transposase-accessible chromatin sequencing (ATAC-seq) and chromatin immunoprecipitation sequencing (ChIP-seq) techniques enable high-resolution mapping of chromatin accessibility associated with viral latency, lytic reactivation, and host transcriptional remodeling [102,103]. EBV has been extensively studied for its impact on cellular chromatin accessibility and DNA methylation using these techniques [103,104,105]. In B cells, ATAC-seq and ChIP-seq were used to show that EBV alters host chromatin accessibility during infection, particularly in regions related to B cell activation and proliferation [103]. EBV latent genes (EBNA1, EBNA2, and EBNA3C) were enriched in different ATAC clusters, suggesting distinct roles in chromatin remodeling during B cell transformation. Reduced Representation Bisulfite Sequencing (RRBS) analysis of EBV-infected normal oral keratinocytes (NOKs) showed that loss of the viral episome led to the hypermethylation of over 13,000 CpG residues in the host, which disrupted gene expression and delayed normal cell differentiation [106]. A multi-omics study integrated whole-genome bisulfite sequencing, ATAC-Seq, and whole-exome sequencing (WES) in NPC to investigate EBV-associated epigenetic dysregulation in the host and found two epigenomic subtypes: one with global hypermethylation and another with global hypomethylation [104]. Both subtypes shared a CpG island methylator phenotype, commonly seen in EBV-associated cancers. The loss of the CTCF host protein, a key chromatin organizer, was linked to methylation changes, indicating that EBV disrupts chromatin boundaries to promote epigenetic reprogramming and immune evasion. In BL, EBV infection alters host DNA methylation in cancer development genes, including ID2 or TWIST and telomerase reverse transcriptase, supporting the role of EBV in driving lymphoma through epigenetic dysregulation [107]. The ChIP-seq experiments in B cells mainly examined histone methylation marks such as H3K9me3 and H3K27me3 on the EBV genome [108]. Loss of these silencing marks opened EBV viral chromatin and caused de-repression of latent and lytic EBV genes.

In NPC, methyl-capture and bisulfite amplicon sequencing identified over 150 hypermethylated CpG islands in host gene promoters, which silenced tumor suppressor genes like ITGA4, RERG, and CR2 [109]. Targeted bisulfite sequencing using hybridization capture followed by NGS was performed across multiple EBV-associated cancers and found cancer-specific methylation patterns in the EBV genome, such as hypermethylation of RPMS1 in GC and BILF2 methylation in NPC and NKT-cell lymphoma [110]. EBV reshapes both host and viral DNA methylation patterns to promote long-term persistence and transformation [111]. The Infinium MethylationEPIC BeadChip identified methylation of promoter regions of host genes involved in chemoresistance (ABCG2, TP73, and BCL2) in EBV-associated GC, while RRBS and ATAC-seq showed that treatment with a demethylating agent (DCB) led to global EBV genome hypomethylation, increased viral chromatin accessibility, and induction of lytic reactivation, revealing potential therapeutic vulnerabilities [105,111]. ChIP-seq, Hi-C, and HiChIP revealed that while host CTCF binding is conserved across EBV latency types, chromatin loop formation varies, bringing distal viral enhancers into proximity with specific promoters [112]. These loops link viral regulatory elements to host oncogenes, and the host chromatin remodeler PARP1 contributes to maintaining this EBV-associated genome architecture.

ChIP-on-chip analysis and sequential ChIP assays revealed that KSHV genomes carry repressive histone marks (H3K27me3, H3K9me3) during latency and activating marks (H3K4me3) during reactivation [96,113]. These transitions are driven by both host enzymes and viral regulators like RTA. The repression of lytic genes by host polycomb repressive complexes 1 and 2 was further validated using ChIP, establishing a clear link between chromatin dynamics and viral latency. ChIP-seq analysis and ChIP-on-chip assay during KSHV infection showed that LANA binds to both latent and lytic gene promoters and recruits host complexes hSET1 and KDM3A to modulate histone methylation, emphasizing the role of LANA in establishing KSHV latency through epigenetic modifications [114,115]. Additionally, ChIP-seq analysis showed that ZIC2, a KSHV RTA host substrate, maintains H3K27me3 at the RTA promoter, thereby maintaining latency, while its loss shifts the chromatin toward an active state, promoting reactivation [116]. Beyond histone modifications, DNA methylation at the RTA promoter also plays a key role in regulating KSHV latency [117]. MAPit single molecule foot printing and bisulfite sequencing were used to identify distinct latency states: a more “closed” chromatin state and a “primed” state, explaining how KSHV balances latency with occasional reactivation, supporting a model where infrequent lytic reactivation ensures the virus’s long-term persistence. More recent high-resolution genomic approaches, including CUT&RUN (Cleavage Under Targets and Release Using Nucleus), which maps protein-DNA interactions by targeted cleavage and sequencing; Capture Hi-C, which detects three-dimensional chromatin interactions; and DNA-FISH analyses on PEL cell lines, demonstrate that host CHD4 acts as a key chromatin repressor of KSHV by interacting with LANA and host chromatin to restrict RNA polymerase II recruitment and maintain latency, while viral lncRNA PAN promotes reactivation by sequestering CHD4 and relieving transcriptional repression [63]. Additionally, CUT&RUN and GroSeq showed that the KSHV terminal repeat (TR) regions act as a viral enhancer during latency-to-lytic reactivation [118]. TR is enriched for activating histone marks bound by LANA with chromatin modifiers, boosting lytic promoter activity. Unlike cellular enhancers, the function of KSHV TR enhancers is largely orchestrated by LANA, demonstrating a virus-specific chromatin regulatory mechanism. CUT&RUN sequencing examined histone modifications of host MYC enhancer regions in PEL cells and identified VGN50-mediated inhibition of coactivator complex recruitment and reduction in H3K27Ac modification [119]. VGN50 is a peptide molecule that mimics the function of K-RTA and down-regulates MYC-mediated gene transcription during lytic KSHV infection.

Early ChIP-seq studies on MHV-68-infected cells demonstrated that histone acetylation, rather than DNA methylation, at the RTA promoter plays a pivotal role in latency-lytic transitions [98]. This observation highlighted RTA as a master regulator of reactivation in MHV-68. Subsequent studies revealed the dynamic association of histone H3 with the promoter and origin of lytic replication during lytic replication, further emphasizing the importance of chromatin remodeling in viral replication [120]. ChIP-seq demonstrated that host polycomb repressive complexes restrict viral gene expression and maintain latency in both KSHV and MHV-68 [121].

## 3. Transcriptomics

The field of transcriptomics, the study of all RNA molecules transcribed in a particular cell or tissue, originated in 1976 after the complete transcriptome sequencing of the bacteriophage MS2 [122]. By 1995, serial analysis of gene expression was introduced as the first technique capable of profiling the transcriptome through chemical tag-based methods in the human pancreas [123]. Microarray technology emerged as an important intermediate approach, allowing large-scale gene expression profiling [124]. Bulk RNA sequencing (bulk RNA-seq) became the dominant method for transcriptomic studies, enabling comprehensive analysis of mRNA libraries (excluding rRNA) with high resolution and sensitivity [125]. Single-cell RNA sequencing (scRNA-seq), first performed in 2009, is a more advanced method in comparison to the bulk RNA-seq technique [126]. scRNA-seq identifies gene expression profiles at the single-cell level, providing an advantage over the issue of cell heterogeneity within a population [127]. The high sensitivity of scRNA-seq allows for the analysis of minimal biological material while allowing depth of resolution for higher detection and transcriptional profiling of rare cell populations. Spatial transcriptomics (ST), introduced in 2016, enables the generation of gene expression profiles and their localization within tissue samples while protecting the integrity of the tissue microenvironment [128,129]. Overall, transcriptomics facilitates differential gene expression analysis under biological conditions, enables novel gene discovery, reveals alternative splicing events, and identifies the roles of non-coding RNAs (Figure 2).

### 3.1. Bulk Transcriptomics

#### 3.1.1. Latent Transcriptomics

EBV, MHV-68, and KSHV demonstrate both unique and overlapping transcriptional strategies during different stages of infection, as revealed through various bulk RNA-seq approaches [17,130,131,132]. Bulk RNA-seq has shown that EBV preferentially infects B lymphocytes and drives them towards differentiation to generate lymphoblastoid cell lines (LCLs) [130,133]. Multiple EBV latency programs defined by distinct viral gene expression patterns in the host cell allow the virus to balance long-term persistence [134]. Bulk RNA-seq of EBV-infected resting human B lymphocytes revealed a global reprogramming of the transcriptome during the pre-latent phase of infection (the initial stage following EBV entry into naïve/resting B cells, preceding classical EBV latency programs) with an upregulation in genes involved in cell cycle progression, specifically G1/S transition, DNA replication and repair, and mitotic nuclear division [130,131]. While EBV latency genes were upregulated as early as two days post infection, with latent membrane proteins 1 and 2A increasing in expression as the infection progressed and cells settled in EBV type III latency, lytic genes were observed to be upregulated two weeks post infection [131]. Transcriptional profiling of EBV-associated angioimmunoblastic T-cell lymphoma using bulk RNA-seq revealed uniform EBV latency II with predominant expression of Bam-HI A rightward transcripts, indicating the absence of a complete lytic cycle [135]. Although the early lytic genes *BNLF2a/BNLF2b* were upregulated, the overall expression pattern remained consistent with latency, suggesting that these lytic transcripts were expressed primarily to facilitate immune evasion rather than productive viral replication. Additionally, using bulk RNA-seq, matrix metalloproteinases (MMPs) have been shown to have capabilities in maintaining latency, with an upregulation in EBV-infected peripheral blood mononuclear cells (PBMCs) and a downregulation in NOK cells [136]. RNA-seq analysis on EBV-infected B cells determined how methionine restriction alters viral and host transcription [108]. Host transcriptional programs associated with one-carbon metabolism, methylation pathways, and B-cell growth were significantly altered.

A comprehensive annotation of the KSHV genome was established using NGS approaches, including mRNA-Seq and ribosome profiling, highlighting the complexity of KSHV latency by identifying novel small ORFs, upstream ORFs, and regulatory strategies like alternative splicing and RNA editing in the host [17]. The discovery of viral PAN RNA interaction with ribosomes during latent KSHV infection suggests potential roles in translational regulation, even in a phase dominated by nuclear viral transcripts. Bulk RNA-seq of KSHV latent infection revealed induction of hypoxia-like transcriptional programs, including upregulation of hypoxia-responsive genes (BIRC3 and NEAT1) to promote cell survival, apoptosis resistance, metabolic reprogramming, and immune evasion [137]. A bulk RNA-seq transcriptomic comparison of KS lesions conducted in endemic and epidemic contexts uncovered significant dysregulation of immune and tumor-related genes during KSHV latency [138]. These findings underscore the virus’s reliance on metabolic and immune pathways for maintaining latency.

DNA microarray analysis identified latency-associated genes in MHV-68, including M2, M11, 73, 74, and 75, in cell culture and infected tissue, with ORF 75 detected for the first time during latency, highlighting the potential for alternative splicing and transcriptional activity during this phase [139]. Additionally, during latency, six novel MHV-68 miRNAs were identified, and their expression, along with the nine known miRNAs, was systematically analyzed, providing insights into miRNA roles during persistent infection [140].

#### 3.1.2. Lytic Transcriptomics

Furthermore, bulk transcriptomic analysis of EBV, MHV-68, and KSHV has also revealed distinct yet overlapping mechanisms of viral and host gene regulation during lytic reactivation [41,136,140]. Bulk RNA-seq analysis reveals that EBV infection induces a global reduction in host cellular transcripts while upregulating viral gene expression [136]. This shift is supported by structural changes in the host genome, including the loss of chromatin loops and increased chromatin accessibility, which collectively favor virion production. Additionally, gene expression profiles differ significantly between different keratinocytes and PBMCs. Bulk transcriptomic analysis of natural killer T-cell lymphoma revealed distinct EBV expression profiles compared to other EBV-associated cancers, with lytic genes such as BBRF3, BLRF2, and BSRF1 significantly upregulated compared to other cancers, reinforcing the presence of disease-specific EBV expression profiles [141]. Multi-platform transcriptomics uncovered a comprehensive EBV lytic transcriptome, detecting novel EBV lytic transcripts, alternative splice isoforms, and multigenic transcripts, suggesting new layers of translational regulation, though the functions of these novel multigenic transcripts remain to be defined [142]. Most recently, bulk RNA-seq was applied to the tumor microenvironment (TME) in EBV-infected, undifferentiated nasopharyngeal carcinoma derived from patients [143]. Further analysis identified three NPC clusters with distinct viral expression and immune environments: the first characterized by late lytic gene expression and immunosuppressive cell recruitment; the second by latent gene expression, impaired antigen presentation, and reduced IFN-β activity; and the third by immediate and early lytic gene expression, indicative of abortive lytic activity facilitating immune evasion and immunosuppression [144]. Comparative RNA-seq performed on HEK-293T cells transfected with viral endonuclease from KSHV, MHV-68, EBV, and HSV-1 indicated a significant decrease in host transcript levels [145].

In parallel, bulk transcriptomic studies of KSHV during the lytic phase also highlight strategic gene regulation mechanisms. KSHV regulates host gene expression during the lytic phase, including mechanisms like host mRNA degradation (host shutoff) to prioritize viral translation [17]. These pathways support KSHV replication and immune evasion, ensuring efficient lytic gene expression. Using bulk RNA-seq, KS lesions were categorized into latent, lytic, and mixed expression profiles [138]. Lytic gene expression was associated with pronounced host angiogenesis and glycolysis transcripts, aligning with the metabolic reprogramming required for active viral replication and tumor growth. Bulk RNA-seq was performed on de novo KSHV infection of human PBMCs, monocytes, and endothelial cells at different time points [146]. The viral gene expression, while similar in all cell types, suggested that KSHV gene expression patterns and latency establishment are cell type specific, with monocytes completely silencing viral gene expressions, except LANA, at 24 h of infection and the human PBMCs and endothelial cells showing detectable levels of PAN RNA and ORF58/59 transcripts along with LANA after 48 h post-infection. Analysis of host and KSHV gene expression signatures of skin and gastrointestinal tract (GI) detected ORF75 in 91% of KS lesions using bulk RNA-seq [147]. Further host gene analysis identified 370 and 58 unique differentially expressed genes in skin and GI KS lesions, respectively, when compared to normal tissues. Interestingly, bulk RNA-seq of BCBL-1 cells treated with VGN50, a K-Rta-derived antitumor peptide, showed a significant downregulation in interferon pathways and genes associated with MYC [119].

Transcriptomic analysis during MHV-68 lytic infection, using DNA microarrays, demonstrated dynamic gene expression patterns during lytic replication, with several early viral genes involved in DNA replication expressed at 8–12 h post infection, while virion structural proteins peak later around 12–24 h post infection, aligning with lytic replication kinetics [139]. During lytic infection, via 454 sequencing, it was shown that cellular miRNAs, including mmu-mir-15b and mmu-mir-16, were highly upregulated during MHV-68 infection, suggesting their involvement in regulating viral and host processes during infection [140]. The viral helicase-primase complex genes in MHV-68 and KSHV exhibit late expression, suggesting a potential role in later phases of the viral life cycle, distinct from the early kinetics seen in other herpesviruses like HSV. Global cDNA array analysis of in vitro MHV-68 lytic infection demonstrated individual transcription profiles of genes, gene hierarchy, and gene transcription kinetics [41]. RNA-seq analysis of HEK-293T cells in the presence of MHV-68 protein muSOX demonstrates a downregulation of cellular mRNAs, showing how MHV-68 suppresses host cell gene expression during lytic infection [42]. The disruption of pervasively transcribed, non-ORF regions of the MHV-68 genome was discovered using RNA-seq analysis, which showed that strand-specific antisense oligonucleotide gapmer probes decreased viral protein expression, suggesting these noncoding segments may serve a functional importance in viral protein expression [43]. A recent temporal bulk RNA-seq study during lytic MHV-68 infection of NIH 3T3 cells profiled host transcriptional changes at 4, 8, 12, and 24 hpi and revealed progressive host gene expression changes throughout infection [148]. In addition to robust induction of innate immune and interferon-response pathways and several metabolic pathways, the analysis identified induction of the pentose phosphate pathway and several epigenetic regulators, including TET2, which promotes DNA demethylation and chromatin accessibility. Functional studies demonstrated that the inhibition of either the pentose phosphate pathway or TET-mediated epigenetic remodeling reduced infectious virus production, revealing a host metabolic-epigenetic crosstalk that supports productive gammaherpesvirus replication.

### 3.2. Single-Cell RNA Sequencing

Single cell RNA sequencing (scRNA-seq) enables the resolution of cellular heterogeneity by capturing transcriptomic differences at the individual cell level [126]. This approach has proven valuable in studying virus-infected populations, where cells can exist in distinct functional states during infection and reactivation.

During EBV infection, scRNA-seq has delineated distinct cell clusters corresponding to latent, early lytic, and fully lytic phases. Latent clusters enriched for super-enhancer-regulated genes were identified, and disruption of these regulatory regions was sufficient to lead to viral reactivation, underscoring their key role in maintaining latency [149]. Lymphoblastoid cell line (LCL) heterogeneity is influenced by immunoglobulin isotypes and viral replication status [150]. In NPC, it is revealed that intra- and interpatient heterogeneity in epithelial–mesenchymal transition states is linked to tumor aggressiveness [151]. Immune dysregulation during EBV infections has been reported, including interferon signaling differences and immune checkpoint upregulation in hemophagocytic lymphohistiocytosis (HLH) and infectious mononucleosis (IM) [152]. Additionally, it is noted that there are altered immune cell populations in EBV-associated GC as well [153]. Regarding KSHV, scRNA-seq was used to uncover transcriptional heterogeneity among latent and lytic viral states in PELs [154]. A 3D air-liquid interface culture model developed to study KSHV infection in oral epithelial cells identified novel intermediate latent states linked to viral reactivation using scRNA-seq [155]. Preferential KSHV infection of specific monocyte subpopulations creates a shift in immune modulation, while distinct infected endothelial populations and their transcriptomic changes post-treatment were identified when KS tumors were profiled [156,157]. scRNA-seq analysis on pediatric donors with EBV-IM and EBV-HLH identified activation of CD8+ T in response to EBV infection compared to healthy donors [158]. However, in HLH patients, weakened humoral immunity was suggested due to decreased plasma cells, contrasting with the enriched plasma cells in IM groups.

### 3.3. Spatial Transcriptomics

Spatial transcriptomics (ST) was developed, encompassing the pre-existing capabilities of scRNA-seq while preserving the cellular environment, bridging a gap in how infected host cells interact with surrounding tissues [128]. Key advances in ST include the ability to pinpoint hybridized mRNA within tissue, predict the positioning of transcripts, and access intraregional transcriptional diversity. Currently, technologies such as Stereo-seq, laser capture microdissection (LCM), Lariat-seq, and Slide-seq have been employed to produce ST data [159]. Among these, Stereo-seq is the latest development, which anchors cyclized DNA “nanoballs” from target tissue onto high-density chips and thus offers high subcellular resolution and large-area capture [160]. Contrastingly, LCM isolates specific tissue regions using laser beams for RNA extraction, permitting a convenient circumvention of RNA purification [161].

Since the introduction of ST, it has frequently been used in conjunction with scRNA-seq, as they complement one another in validating disease mechanisms. In EBV research, stereo-seq combined with scRNA-seq in NPC revealed a diversity of cell types within tertiary lymphoid structures (TLS), including clusters of immune cells localized near inflammatory sites [162]. This enabled spatial mapping of interactions between assorted plasma cell subsets and EBV-malignant cells, suggesting TLS may modulate immune cell differentiation, antibody production, and trafficking. Another study focused on PD-1/PD-L1 axis (programmed cell death protein-1 and its ligand)-mediated inhibition of immune responses in TME of EBV-positive diffuse large B-cell lymphoma, where scRNA-seq revealed reduced immune activity, TME heterogeneity, and the mechanism of response to PD-1 blockade [163]. ST provided precise localization of PD-1/PD-L1 expression and highlighted major differences in the types and spatial distribution of 12 immune cell clusters between EBV-positive and EBV-negative tumors. These findings using ST have opened new avenues for medicinal consideration in numerous EBV-associated diseases, many of which manifest in inconsistent ways that lead to poor prognosis. ST on KS skin tumors from clinical trial participants determined seven gene expression clusters, two of which expressed ORF72 and K12 KSHV latent genes, PAN lytic KSHV gene, as well as Stanniocalcin 1 (STC1) and Fms-related tyrosine kinase 4 (FLT4) [164]. STC1 and FLT4 had significantly altered expression in KS tumors, including genes involved in angiogenesis, extracellular matrix remodeling, cell adhesion, and neural development. Published ST studies on gammaherpesvirus pathogenesis remain scarce due to its recent development and resource-intensive platforms. To date, no studies have employed ST in MHV-68 research, highlighting a crucial gap in the modeling of gammaherpesvirus biology.

### 3.4. Novel and Advanced Sequencing Techniques

High-throughput sequencing technologies have transformed our understanding of the complex transcriptomes of GHVs. While NGS, including standard RNA sequencing, provides broad gene expression profiles, recent innovative methods have gone beyond by precisely mapping intricate features of viral transcripts at single-nucleotide resolution [142]. Techniques like Cap Analysis of Gene Expression sequencing (CAGE-seq), deepCAGE, and polyadenylation sequencing (pA-seq) have annotated overlapping transcription of the EBV genome [142,165]. A multiplatform transcriptomic study was pivotal in identifying 322 transcription start sites (TSSs), 57 transcriptional end sites (TESs) and 351 polyadenylation sites across EBV viral genomes, out of which 145 TSSs, 12 TESs and 241 polyadenylated transcripts were novel identifiers of this study. These sites revealed pervasive alternative splicing and polycistronic transcripts that were previously difficult to resolve [142]. Specialized approaches, such as RNA Annotation and Mapping of Promoters for the Analysis of Gene Expression (RAMPAGE), used to understand reactivation of KSHV in iSLK.219 cells and the PEL cell line TREx-BCBL1-RTA, have further enhanced promoter mapping by selectively capturing capped 5′ RNA ends, uncovering novel and cell-type-specific TSS clusters during KSHV viral reactivation [166]. The integration of short-read Illumina sequencing with long-read platforms, including PacBio SMRT and Oxford Nanopore sequencing, has enabled full-length characterization of viral transcripts. This multi-platform approach has identified hundreds of novel transcripts, splice variants, and regulatory elements critical for understanding viral latency, lytic reactivation, and immune evasion [167]. Long-read sequencing has particularly illuminated the diversity of viral antisense transcripts and uncovered previously unrecognized RNA isoforms, highlighting functional complexity that short-read sequencing alone cannot fully capture [168]. Thiol (SH)-linked alkylation for the metabolic sequencing of RNA (SLAM-seq) is a high-throughput RNA sequencing method that distinguishes newly synthesized RNA from pre-existing RNA by detecting 4-thiouridine (s4U) incorporation in RNA species at single-nucleotide resolution [169]. SLAM-seq profiled BC-1 and BCBL-1 cells in the presence of VGN50 and the BET bromodomain inhibitor, JQ1 [119]. It showed a significant downregulation of host genes in both cell types, demonstrating that KSHV K-RTA reduces host cell gene transcription during lytic infection. Despite challenges such as overlapping transcripts and technical biases, advancements like deepCAGE and computational tools have improved sensitivity, allowing for the detection of low-abundance transcripts and accurate quantification of overlapping viral genes [132,170]. Together, these complementary sequencing technologies provide an unprecedented resolution of herpesvirus transcriptomes, advancing our knowledge of viral gene regulation and host–virus interactions while opening new avenues for therapeutic development.

## 4. Proteomics

Proteomics has revolutionized gammaherpesvirus biology by enabling the comprehensive identification and quantification of viral proteomes, the characterization of host and viral post-translational modifications (PTMs), and the mapping of complex host-viral protein–protein interaction (PPI) networks [22,31,37,44,171,172,173,174]. These technical advancements provide critical insights into the molecular mechanisms governing viral replication, latency, and host pathogenesis [175]. Traditional protein methods, such as Western blotting, immunoprecipitation (IP), and enzyme-linked immunosorbent assays (ELISA), established the backbone for proteomics analysis [161]. Other traditional assays like the yeast two-hybrid assay (Y2H), while valuable for PPI studies, showed high instances of error while proving to be expensive and time-consuming to expand. Although these traditional assays continue to play a critical role in validating experimental findings, their low throughput underscores the need for modern high-throughput proteomic platforms capable of capturing the complexity of viral and host proteomes.

**Figure 3:**
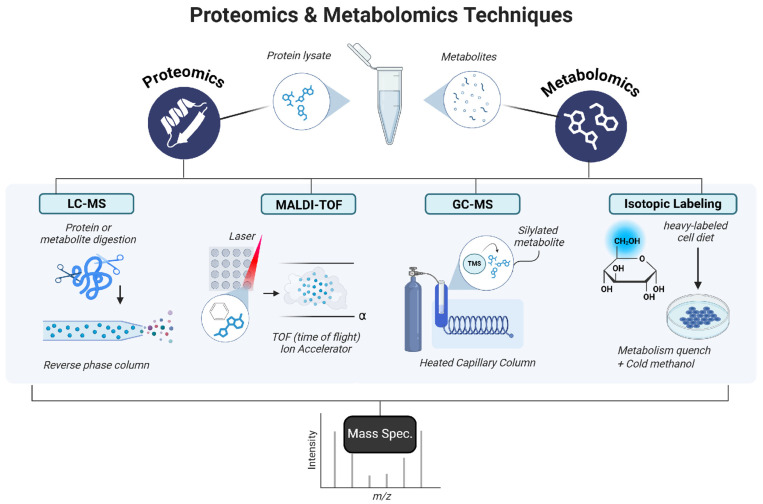
Metabolomics and proteomics techniques (left to right) LC-MS, MALDI-TOF, GC-MS, and isotopic labeling. All techniques are performed on both proteins and metabolites except for GC-MS, which is ideal for metabolites only due to size restrictions for silylation. Created in BioRender. Lab, S. (2026) https://BioRender.com/l9f2o2d (accessed on 8 June 2026).

Mass spectrometry-based proteomic approaches enable large-scale, unbiased protein identification and quantification of thousands of proteins, providing a global view of the viral and host proteome [176]. Affinity purification-mass spectrometry (AP-MS), which isolates protein complexes via tagged bait proteins coupled with MS analysis, has been widely applied to define herpesvirus-host PPI networks [22,177,178]. Advanced MS technologies, including tandem MS (MS/MS), label-free quantification, and isotope labeling techniques like Stable Isotope Labeling by Amino Acids in Cell Culture (SILAC) or Tandem Mass Tag (TMT), enable the detailed characterization of the dynamic interplay between viral-host protein interactions [179,180]. Liquid chromatography-tandem mass spectrometry (LC-MS/MS), coupled with high-performance liquid chromatography (HPLC) or ultra-high-performance liquid chromatography (UHPLC), provides high-resolution separation and sensitive detection of proteins and PTMs during GHV infections [173,181,182]. Complementary platforms such as matrix-assisted laser desorption ionization–time of flight mass spectrometry (MALDI-TOF) offer rapid protein detection with exceptionally short run times [182,183]. Moreover, high-throughput proteomics technologies, including isobaric tags for relative and absolute quantitation (iTRAQ) and protein microarrays integrated with bioinformatics, further delineate altered signaling pathways and protein networks, clarifying the molecular mechanisms underlying GHV-associated diseases [174,184,185] (Figure 3).

### 4.1. Viral Proteomics

Proteomic analysis of GHVs has identified structural and regulatory viral proteins, elucidating their roles in viral replication, immune evasion, and disease pathogenesis [31,180]. The proteome of the mature EBV virion was first characterized in 2004 using LC-MS/MS, revealing a composition that was largely homologous to the herpes virion [31]. Among them, identified components included tegument proteins (BLRF2, BRRF2, BDLF2, and BKRF4) involved in assembly, egress, and host machinery manipulation, as well as envelope glycoproteins (gp350, gH/gL, and gM/gN) that mediate attachment, membrane fusion, and envelopment. Several host proteins, including actin, cofilin, tubulin, and heat shock proteins (HSP70/90), were also incorporated into the virion. Later, the tegument protein complex BBRF2-BSRF1, involved in tethering the EBV to the Golgi apparatus, envelopment, and egress, was discovered using UHPLC-MS and MALDI-TOF MS [182]. Disease-specific EBV proteomic profiling employing iTRAQ-coupled 2D LC-MS/MS for NPC identified 12 upregulated proteins associated with cytoskeleton formation, metabolic pathway dysregulation, DNA binding, cell proliferation, apoptosis, and signal transduction, with alterations in the p53 and NF-κB signaling pathways, and provided a PPI network between EBV and NPC proteins [186]. EBV has also been associated with IM and HLH [187,188]. In EBV-HLH, UPLC-MS/MS analysis of exosomes detected immune dysregulation markers such as C-reactive protein, moesin, galectin-3-binding protein, and heat shock protein as well as impaired liver function and coagulation markers such as plasminogen and fibronectin [180]. Comparative LC-MS profiling of EBV-IM and EBV-HLH plasma revealed strong enrichment of the complement system, with 63, 18, and 11 proteins upregulated in IM, HLH, and both groups, respectively [189]. These proteins mapped to pathways involving complement activation, platelet degranulation, fibrin clot formation, endocytosis, and kinase-regulated signaling. Moreover, in EBV-positive classical HL, protein microarray analysis revealed population-specific antibody signatures, with 14 IgG antibodies in the East Asian profile linked to European descent, 15 antibodies (2 IgA, 13 IgG) linked to Asian descent, and a shared core set of five anti-EBV IgG antibodies (LMP-1, TK, BALF2, BDLF3, and BBLF1) [184].

The first KSHV virion proteome was reported in 2005, identifying several viral and cellular proteins associated with the viral structure via MALDI-MS and LC-MS/MS [37,38]. UHPLC-LC-MS expanded the known KSHV proteome by identifying 17 additional viral proteins, including 10 novel factors linked to lytic reactivation, lytic replication, genome replication, proliferation, immunomodulation, and immune evasion, as well as glycoproteins and three viral interferon regulatory factors that suppress type I and II interferon responses [181]. Beyond structural profiling, LC-MS/MS has been employed to identify host factors that enhance KSHV infectivity during cellular senescence [190]. Comparative surface proteomics of senescent endothelial cells revealed the significant upregulation of host membrane proteins, including caveolin-1, integrin α2, F11R, and CD109. This remodeling of the membrane proteome creates a more permissive environment for viral binding and entry, linking age-related cellular changes to increased KSHV susceptibility. Furthermore, using Proteomics of Isolated Chromatin fragments (PICh), a targeted chromatin-capture method used to identify and quantify proteins associated with specific genomic loci, followed by MS in KSHV-positive BJAB cells, latent KSHV infection was shown to reshape the telomeric proteome [191]. While the core shelterin complex, a hexameric assembly that caps telomeres to suppress DNA damage signaling, is enriched in both parental and KSHV-positive BJAB cells. Similarly, MHV-68 was profiled for the first time in 2003 using LC-MS/MS analysis of extracellular virions, identifying 14 capsid, tegument, and envelope proteins [44]. This was later expanded using 1D gel/nano LC-MS/MS analysis of extracellular MHV-68 virions, identifying 31 structural proteins [192]. Later, MALDI-TOF MS and tandem micro-LC-MS/MS analysis of the bronchoalveolar lavage in MHV-68-infected mice demonstrated enrichment of oxidative stress-associated proteins, a potential salient feature of MHV-68 pathogenesis [193].

### 4.2. Post-Translational Modifications

PTMs play essential roles in GHV biology, where MS-based studies have revealed how these chemical modifications of host and viral proteins enable GHVs to manipulate cellular pathways and promote infection [18,171,172,173,179,194,195,196]. Major PTMs implicated in GHV infection include acetylation, phosphorylation, glycosylation, ubiquitination, and others.

Protein acetylation, mediated by lysine acetyltransferases using acetyl-CoA, regulates both histone and non-histone proteins during GHV infection [171,197,198]. TMT LC-MS/MS and enrichment analysis reveal that KSHV extensively reprograms the host non-histone acetylation proteome to drive tumorigenesis [171]. Specifically, the deacetylation of SERPINE1 mRNA binding protein 1 (SERBP1) facilitates viral transformation by modulating ferroptosis, establishing SERBP1 as a critical regulator of the KSHV-mediated oncogenic program.

Phosphorylation, the addition of a phosphate group to the protein, is critical in both the early and late stages of GHV infections [199]. Phosphoproteomics includes the study of protein phosphorylations and their implications in cellular functions. In EBV, a SILAC-based LC-MS/MS analysis revealed the viral kinase BGLF4 hyperphosphorylates over 1000 cellular proteins involved in the DNA damage response (DDR), mitosis, and cell cycle regulation during lytic replication in Akata cells [179,200]. An LC-MS/MS analysis of EBV-infected primary B cells revealed a unique temporal protein expression profile, including the phosphorylation of the host ZFP36 family, the mitogen-activated protein kinase (MAPK) pathway, and RNA-binding proteins, which confirmed that the activation of STAT3 and p38/MK2 is essential for the activation of viral protein EBNA2, facilitating IL-6 and TNFα release and BRF1 activation [201]. In KSHV, iTRAQ-based HPLC-MS/MS analysis of the phosphoproteome and proteome of KSHV-infected endothelial cells revealed alterations in the carbon, lipid, and hypoxia-induced factor signaling pathways during latency [172]. Similarly, LC-MS/MS phosphoproteomic analysis of KSHV-infected epithelial cells revealed that host protein phosphorylation is widely affected by the induction of lytic replication [202]. Viral protein ORF45 activates p90 ribosomal S6 kinase, which is required for lytic reactivation and virion production, resulting in the upregulation of nuclear proteins. LC-MS/MS analysis revealed that KSHV vCyclin recruits CDK6 to phosphorylate the carbamoyl-phosphate synthetase 2, aspartate transcarbamoylase, and dihydroorotase (CAD) protein, a key enzyme of the de novo pyrimidine synthesis [203]. Likewise, nano-LC-MS/MS and HPLC-coupled high-resolution MS/MS analyses of MHV-68 identified alterations in host phosphoproteins and viral proteins, with predominant MAPK and CDK signaling events [173]. Functional studies revealed that ERK1/2 and CDK1/2 activities facilitate viral replication, while the v-cyclin D ortholog contributed directly to this MAPK/CDK signature. These findings highlight how MHV-68 hijacks signaling networks to promote infection and optimize its replication program.

Glycosylation refers to the addition of glycans, small carbohydrate molecules, to the protein and is also a target of GHVs [204,205]. Glycosylation assists viral infection by enhancing host cell attachment, internalization, and regulation of immune responses [206]. Nano-LC-MS/MS mapping of the O-glycoproteome of EBV in human BL B cells revealed the O-glycosylation of six EBV envelope glycoproteins, including gB, gN, gp42, gp78, gp150, and gp350 [18]. Comparative analyses of herpesvirus proteomes, including EBV, varicella-zoster virus (VZV), and human cytomegalovirus (HCMV), further uncovered widespread O-glycosites on viral envelopes and conserved O-glycan patterns on distinct homologous proteins, underscoring the evolutionary importance of glycosylation for host interaction and immune evasion across the herpesvirus family [18].

Ubiquitination represents an additional layer of viral control over host cell pathways by modulating host protein functions for maximal viral replication [207]. A SILAC LC-MS/MS study revealed dynamic changes in EBV protein ubiquitination after IgG crosslinking-mediated antigen-induced B cell receptor activation, a viral reactivation trigger in Akata BL cells [194]. In parallel, studies on deubiquitinating enzyme (DUB) activity during EBV latency demonstrated its role in maintaining the viral life cycle [208]. MS/MS analysis identified the upregulation of several active DUBs in BL. A unique DUB activity was identified after EBV latency establishment and correlated with changes in cell proliferation rates, suggesting its importance in establishing cell transformation. Furthermore, the addition of antigen F (HLA-F) adjacent transcript 10 (FAT10), a ubiquitin-like protein upregulated during KSHV infection, is known as “FAT10ylation” [209]. The first report on the contribution of FAT10ylation for effective virus infection and virion production identified FAT10ylated proteins (e.g., ORF59 and ORF61) through LC-MS/MS analysis. Upregulation of the UBE1L2-FAT10 system during KSHV lytic replication suggests the importance of this modification in KSHV pathogenesis. UFMylation refers to the covalent attachment of ubiquitin-fold modifier 1 (UFM) to other proteins and has been implicated in immune evasion strategies during DNA virus infection [210]. LC-MS/MS analysis mapped EBV-EBV and EBV-host PPI in lytic B cells and revealed the inhibition of NLRP3 inflammasome activation through the viral protein BILF1-dependent MAVS UFMylation [211]. BILF1 blocks NLRP3 to prevent pyroptosis and sustain lytic replication while avoiding immune detection. Lastly, SUMOylation is a PTM where the SUMO (small ubiquitin-related modifier) conjugates the lysine residues of a target protein, regulating its activity, localization, and protein stability [212,213]. An LC-MS/MS analysis of EBV-GC cells identified seven viral proteins that were SUMOylated in EBV lytic infection, including two new reports (BORF2 and BALF2), as well as revealed phosphorylation and SUMOylation events in the host antiviral TRIM24/28/33 complex during infection [195]. Besides the covalent SUMO modification, proteins can interact non-covalently with SUMO through their SUMO-interacting motif (SIM), allowing the interaction of SUMOylated proteins with their partners [214]. MALDI-TOF MS of the KSHV LANA-SIM interacting complex identified DNA-PKc, CBP, p300, Sin3A, and KAP1 as SUMO-2-associated partners, revealing that KAP1 is the primary poly-SUMO-2-modified target recognized by the LANA-SIM. MALDI-TOF MS analysis of KSHV-positive B lymphocytes identified a unique proteomic interaction profile involving 151 proteins associated with LANA^SIM^ involved in cell cycle regulation, DNA unwinding, DNA replication, and pre-mRNA/mRNA processing [215]. SILAC-based LC-MS/MS analysis of KSHV-infected cells revealed 40 host proteins undergoing RTA-dependent ubiquitination as well as a significant increase in the HSP70 family cellular chaperones at nuclear envelope-associated replication and transcription compartments [196,216]. The study further identified that RTA facilitates KSHV immune evasion by degrading MHC class I components, impairing TAP-dependent peptide transport, and increasing intracellular HLA and proteasome subunit degradation [196]. These results establish RTA as a master proteomic regulator that promotes viral reactivation while shielding KSHV from host immunosurveillance.

### 4.3. Protein–Protein Interaction (PPI)

Early efforts to map PPI in EBV and KSHV began in the early 2000s [217]. A genome-wide PPI map of KSHV generated via Y2H arrays identified 123 interactions, revealing that herpesviruses possess distinct network architectures. Similarly, Y2H assay-based exploration on EBV revealed 43 interactions between viral proteins and 173 between EBV and human proteins [218]. Despite their utility, these early Y2H-based datasets exhibited variable reproducibility; for instance, only approximately 40–50% of the identified interactions were subsequently validated by co-immunoprecipitation (Co-IP) or co-affinity purification (Co-AP) [217,218]. Y2H screening in MHV-68 combined with multiple functional validation approaches created a PPI network spanning 25 viral proteins and 197 cellular proteins [19]. Modern studies of PPIs use MS and its related techniques to grow and clarify specific sections of the understood network. For example, BL subtypes were compared with and without EBV infection using iTRAQ-MS/MS analysis, revealing the unique regulation of 144 cellular proteins [174]. This study identified differential expression of host HSPs across BL variants: HSP90AB1 was upregulated in sporadic BL, whereas HSP90B1 predominated in endemic BL. These findings suggest that HSP90 isoforms serve as subtype-specific molecular signatures, implying that isoform-targeted inhibition could provide a tailored therapeutic approach for distinct BL pathologies [174,219]. Similarly, in KSHV, large PPI studies have created a comprehensive interaction map between the KSHV viral and host proteins using AP-MS that revealed over 50,000 interactions, drawing attention to ORF24 and RNA polymerase II as key to KSHV late gene expression [22]. Further investigation into HSPs during KSHV infection using HPLC-MS and LC-MS revealed the link between KSHV infection therapeutics and its interaction with HSP70 through the HSP70–HSP90 organizing protein (Hop), a promising disruption target during lytic reactivation for potential therapeutics [220]. Using GeLC-MS/MS, 191 cellular proteins were identified in the complex with mLANA in MHV-68 infection, specifically host proteins Hsc70, TRIM21, SRSF6, and SRSF7 [221]. mLANA engages a broad host protein interaction network during lytic replication, and Hsc70 is a critical host factor that supports efficient MHV-68 replication, as demonstrated by SILAC LC-MS/MS.

Subsequent research using TMT-nano-UHPLC-MS/MS identified over 7000 cellular and 71 viral proteins altered during KSHV lytic reactivation, with significant enrichment for host cell components involved in mRNA processing, translation, and protein folding [222]. Recently, a bottom-up IP-MS approach identified an interaction between the KSHV viral protein kinase and the cellular deubiquitinase USP9X [223]. Functional studies further revealed that USP9X depletion inhibits both viral reactivation and the production of infectious virions. By employing the Rapid Immunoprecipitation Mass Spectrometry of Endogenous Protein (RIME) assay in iBCBL1 cells, transient in vivo interactions, and protein complexes associated with RTA on the chromatinized KSHV genome were identified [224]. The RTA transactivator hub was mapped by identifying established viral/chromatin partners and the novel recruitment of the RNF20/40 E3 ubiquitin ligase to promote the lytic cycle. RIME identified 87 proteins that interacted with the KSHV transactivator (K-Rta) sequence, a small peptide sequence where these protein complexes were involved in RNA processing, RNA splicing, chromosome organization, DNA repair, and DNA replication [119]. These studies represent the promising direction of proteomics-based KSHV-host PPI investigation for therapeutic targets.

While in EBV, using BioID combined with high-resolution electrospray ionization tandem mass spectrometry analysis (HR-ESI-MS/MS) revealed over 1000 proteins interacting with EBV LMP1, a widely implicated EBV latency membrane protein, expanding the network created from Y2H assays of the past [225]. The study highlighted the role LMP1 plays in intracellular trafficking and exosome regulation and secretion. Moreover, data-independent acquisition MS and LC-MS identified 137 differentially expressed proteins during EBV-GC and identified several possible hub genes and potential biomarkers for EBV-GC [226]. Analysis of EBV+ or EBV− tumor sample datasets using STRING defined the EBV-host PPI network, identifying 11 hub proteins whose expression signifies increased responsiveness to immunotherapy in GC [227]. Other work in the field using a NanoBiT assay has identified 195 intra-viral PPIs that highlighted BLRF2 as a key tegument network hub protein [228]. Notably, the first lytic cycle proteomic map of EBV-host and EBV-EBV protein interactions using LC-MS/MS identified 1398 viral-host and 90 viral-viral high-confidence interacting proteins, revealing the EBV association with cellular degradation machinery and enrichment of the extracellular exosome and mRNA splicing pathways [211].

While protein–protein interactions (PPIs) are primarily characterized through in vivo and in vitro assays, computational approaches such as ℓ1-norm optimization provide a predictive framework for identifying putative interactomes [23]. Such analyses reveal that despite significant sequence homology, EBV and KSHV exhibit divergent topological architectures. This structural dissimilarity suggests that these gammaherpesviruses are undergoing network-level divergence, hindering the cross-species prediction of interactome dynamics. However, computation analysis is still powerful in developing a map of 51,485 EBV-human functional protein–protein interactions, 869 stringent physical PPIs, and 46,050 relaxed physical PPIs, with 58 confirmed through literature evidence [229]. Another group used bioinformatic tools such as STRING and GeneMania and predicted PPIs of ORF 73 (LANA) with KS-associated human oncogenes, highlighting p53 signaling in host apoptosis and the prognostic marker of KS [185]. The STRING interaction map in KSHV infection determined RNA polymerase II-interacting proteins were increased >1.5-fold in the presence of K-RTA peptide, where overlapping PPIs suggested a pre-assembled large coactivator complex during KSHV reactivation [119]. A modern computation platform, Tapioca, demonstrates how machine learning analysis can advance PPI networks by increasing the strength of networks by consolidating multiple modern MS approaches like thermal proximity coaggregation (TPCA)–MS, cofractionation–MS (CF–MS), and ion-based proteome-integrated solubility alteration (I-PISA), and increasing confidence by aligning multiple networks [230]. The effectiveness of this computational approach was demonstrated by predicting 45,000 to 80,000 PPI per measurement time point during KSHV reactivation, which led to the identification of NUCKS as a hub protein for KSHV replication.

## 5. Metabolomics

Metabolomics provides a holistic view of the host cell metabolome by classifying and quantifying metabolites across multiple complex pathways [231]. Studies have explored the alteration in cellular metabolism, including central carbon pathways, like glycolysis, the TCA cycle, and the pentose phosphate pathway, as well as nucleotide, lipid, and glutamine metabolism, to reveal virus-specific metabolic modulations in the host during viral infection. Metabolomic analysis to identify alterations driven by viral infection can further enhance the understanding of host–pathogen interactions, stemming from the core concept that high-level regulation of biological pathways will be reflected in changes to the metabolome [232]. Furthermore, lipidomics, a sub-branch of metabolomics, involves large-scale analysis of the lipidome, including lipid biosynthesis and metabolic pathways within cells [233]. Recently, it has been established that metabolites and their pathways are central to the immune responses generated by the immune cells to viruses [234]. Metabolomics can help identify infection-associated metabolic targets that impact the pathogen, the host, or both [235,236]. A high degree of specificity is needed to differentiate these small molecules, necessitating specialized techniques like nuclear magnetic resonance (NMR), liquid chromatography-mass spectrometry (LC-MS), and gas chromatography-mass spectrometry (GC-MS). NMR detects relatively few metabolites reliably and rapidly, allowing for the study of unique metabolomic signatures for a single condition or quantifying changes in known metabolic pathways by tracking levels of isotopically labeled ^13^C and ^15^N. Conversely, MS yields a higher sensitivity to metabolites for the measurement of minute differences [235]. MS is combined with techniques such as gas chromatography and liquid chromatography, giving rise to GC-MS for smaller, volatile compounds and LC-MS for larger, polar metabolites [237]. The largest challenges in these approaches come from the complexity of the metabolome, with possibly over 52 different classes of compounds, including different atoms, functional groups, and structures. Moreover, metabolomes in human systems can be highly variable from one sample to the next, person to person, and tissue to tissue. Metabolomic techniques are precise but need a very large, diverse data set to glean meaningful data (Figure 3).

### 5.1. Global Metabolomics

Global metabolomics refers to the study of untargeted cell-wide small molecule changes to uncover biomarkers of disease and to identify regulated biological pathways [39]. The global metabolomics study of herpesviruses is an established branch of bulk study in which articles shed light on changes in the metabolic pattern of infected cells. Untargeted metabolomic (LC-MS/MS) analysis conducted in latently EBV-infected versus mock-infected B cells showed an increase in the NAD de novo biosynthesis pathway via upregulation of indoleamine 2,3-dioxygenase 1 (IDO1), which is required for EBV to establish latency from pre-latency [20]. Both IDO1 and NAD de novo biosynthesis increase initial mitochondrial respiration during early EBV infection, while later ATP generation is a result of glycolysis. A multi-omics approach (ATAC-seq, RNA-seq, and LC-MS/MS) was performed in studying the immortalization of EBV-infected B lymphocytes, cells where EBV is known to establish latency [103]. Both transcriptomic and metabolomic analyses indicated purine metabolism to be significantly activated. Metabolomic analysis (GC-MS and LC-MS/MS) of EBV-associated GC (EBVaGC) revealed significant changes in amino acid and lipid metabolism [238]. Pathways involved in arginine, proline, glycine, serine, threonine, alanine, aspartate, and glutamate metabolism were dysregulated, while lipid metabolism showed increased phospholipids and decreased triglycerides. Plasma lipidomic (LC-MS/MS) analysis was performed on healthy patients and patients with NPC and showed that lipid droplet accumulation is induced due to EBV infection during latency [239]. The lipidomic profile shifts across stages of lytic reactivation, with late lytic infection characterized by a reduction in overall lipid species compared to the early lytic phase or latency. EBV latency is metabolically linked to host methionine and one-carbon metabolism because these pathways provide methyl donors required for epigenetic silencing of the viral genome [108]. LC-MS was used to profile intracellular metabolites involved in the methionine cycle, folate cycle, and SAM-dependent methylation and found that methionine restriction depleted S-adenosylmethionine (SAM) and methyl donor pools in the host cells, which are required to maintain EBV latency.

In KSHV-infected cells, a global metabolomics study utilizing GC-MS and LC-MS explored changes in metabolite levels during latency [39,240]. It was reported that the increase in long-chain fatty acids (LCFA) observed was due to synthesis, and therefore, KSHV induces lipogenesis during latency [39]. KSHV-infected oral epithelial cells have a markedly altered metabolic profile at 4 h of latent infection, revealing a rapid change in the metabolic profile, including the urea cycle, the asymmetrical dimethylarginine (ADMA) and symmetrical dimethylarginine (SDMA) pathways, and de novo pyrimidine biosynthesis [240]. While studying the requirement of spermidine during KSHV latent infection using LC-MS in both 3D and 2D cell cultures, it was shown that KSHV regulates polyamine metabolism, and its dysregulation reduces the integrity of the extracellular matrix [241]. KSHV infection in human keratinocytes revealed changes in various metabolic pathways in the host [203]. A targeted global metabolomics (LC-MS/MS) on KSHV latently infected cells showed a significant increase in glycolytic intermediates and pyrimidine synthesis metabolites. The CAD enzyme, a key enzyme of de novo pyrimidine synthesis, activated pyrimidine synthesis during KSHV infection to meet the requirements of viral and host DNA replication and cell division. Furthermore, it was demonstrated via LC-MS that KSHV-infected 3D cultures require proline biosynthesis as well and enhance tumorigenesis [242]. Pyrroline-5-carboxylate (P5C) reductase (PYCR/P5CR) increases intracellularly during infection by interacting with the KSHV viral type I transmembrane glycoprotein K1. The authors show that K1-induced proline synthesis is responsible for collagen synthesis to enhance growth in 3D cell cultures.

Global metabolomic and lipidomic analysis of MHV-68-infected cells reveals that lytic GHV infection increases host cell glucose, glutamine, lipid, and nucleotide metabolism [45]. Lipidomic analysis (LC-MS) of host cells revealed that lytic MHV-68 infection remodels the host cell lipidome, with peak abundance of triacylglycerides during early lytic infection and peak amounts of free fatty acids and diacylglycerols appearing later during the lytic infection. Furthermore, inhibition of glucose metabolism, glutamine metabolism, and lipogenesis reduced infectious virus production, revealing that host cell metabolic alteration is required for productive lytic infection.

### 5.2. Isotopic Labeling

Stable isotope-resolved metabolomics has become an essential approach in viral infection studies because it enables direct measurement of metabolic fluxes and nutrient utilization, revealing how viruses reprogram host metabolism to support replication, latency, and immune evasion [203,216]. Metabolic flux analysis (U-^13^C-glucose, 2,3,3-^2^H-serine, and U-^13^C-serine) of EBV-infected B cells revealed that serine is the fuel source of EBNA2-induced one-carbon metabolism and that EBV activates de novo serine synthesis [32]. This activation and mitochondrial remodeling are critical to growth and proliferation. In newly EBV-infected primary B-cells, it was found, via isotope tracing of U-^13^C-glutamine and U-^13^C-glucose, that glutamine is the major carbon source sustaining aspartate biosynthesis and maintenance [243]. The upregulation of cardiolipin biosynthesis due to EBV infection is critical to EBV cell transformation, LCL survival, NADPH generation, mitochondria respiration, mitochondrial one-carbon metabolism, and GOT-driven aspartate biosynthesis. Isotope tracing of U-^13^C-tryptophan revealed that de novo NAD biosynthesis occurred in early EBV-infected B cells to fuel 64% of the NAD^+^ pool and part of NADH; transient activation of the kynurenine pathway was utilized to support de novo NAD biosynthesis [20]. Metabolic flux analysis (^13^C-glucose) also revealed that in an EBV-immortalized line, high Myc-expressing cells had significantly increased amino acid uptake, a modest increase in glycolytic pathway flux, increased glutamine consumption, and high mitochondrial metabolism with an increase in the TCA cycle, amphibolic fluxes, and oxygen consumption rate [244]. UHPLC-MS metabolomics using U-^13^C_6_-glucose tracing determined that inhibition of monocarboxylate transporters (MCT1/4) alters host metabolism in EBV- and KSHV-associated lymphomas [245]. Dual inhibition of MCT1 and 4 caused an increase in intracellular lactate and ROS accumulation as well as a decrease in NAD^+^/NADH ratios and cellular glutathione pools, providing mechanistic evidence lactate export is essential for viral lymphoma metabolism.

Recently, it was demonstrated in wild-type (WT) KSHV-infected human oral keratinocytes the incorporation of [U-^13^C] glucose and [Amide-^15^N] glutamine in glycolysis and pyrimidine metabolism, respectively, as compared to mock [203]. There was upregulation of pyrimidine synthesis in WT KSHV in contrast to mutant KSHV lacking vCyclin. Understanding how GHVs exploit host metabolic pathways is essential for identifying metabolic pathways that can be therapeutically targeted. Metabolomics provides a comprehensive view of virus-induced metabolic remodeling under defined conditions, offering mechanistic insights that can support the development of more effective therapeutics.

## 6. Discussion

In this review, we have presented key findings from genomic, transcriptomic, proteomic, and metabolomic studies, illustrating the complex interaction between the host and GHVs, specifically EBV, KSHV, and MHV-68 (Table 1).

Genomic studies, ranging from early Sanger sequencing to modern NGS, have elucidated the genetic diversity of EBV and KSHV along with defining MHV-68 as an experimental model [2,71,83,88]. Genomic diversity noted in latency-associated and structural genes underscores their critical roles in viral pathogenesis [17,130,131,132]. Whole-genome sequencing has revealed geographic variations and diversity within patient populations, identifying the relationship between viral evolution and host factors [79,84]. Comprehensive genomic analyses have provided foundational data to address the GHV-associated diseases [15,97,98,99].

Transcriptomic studies using bulk RNA sequencing (RNA-seq) have unveiled virus-specific adaptations, such as the reliance of KSHV on metabolic reprogramming, contrasted with novel latency-associated genes identified in MHV-68, which illustrate distinct regulatory mechanisms [132,165,168]. Furthermore, single-cell RNA sequencing and spatial transcriptomics have provided insights into cell-to-cell heterogeneity while preserving the spatial resolution, revealing transcriptional diversity that impacts the understanding of viral latency and reactivation [156,163,164]. Together, these transcriptomic tools are pivotal in defining how GVH manipulates host cellular machinery while maintaining their viral transcriptional programs.

Proteomic studies have greatly expanded our understanding of the proteins involved in herpesvirus biology [189,220]. Mass spectrometry has uncovered key structural and regulatory proteins, facilitated the identification of potential biomarkers, and revealed host–virus protein–protein interactions (PPI) [22,174,211,226]. The comparative analysis of these interactions across EBV, KSHV, and MHV-68 opens new avenues for targeted therapeutic interventions. The use of MHV-68 as a model organism allows for comprehensive exploration of GHV proteomes and potential therapeutic targets [173]. Future research should focus on revealing the distinct PPIs linked to various EBV-associated diseases, such as gastric cancer, Burkitt’s lymphoma, and nasopharyngeal cancer, as research is currently limited. Creating detailed PPI networks for KSHV-associated conditions like PEL, KS, and MCD will also provide new insight into the similarities and differences in regulation across these pathologies. Employing cutting-edge proteomic techniques, such as cross-linking mass spectrometry, will be instrumental in unveiling protein interactions in their native environments, pushing forward our understanding of co-infections and the multifactorial nature of EBV- and KSHV-associated diseases.

Metabolomic studies have recently contributed new insights to the critical pathways altered and required during KSHV, EBV, and MHV-68 infections [20,45,242]. Global metabolomics has given us a broad understanding of modified pathways during infection, but stable isotope-resolved metabolomics has provided the opportunity to quantify altered metabolites throughout infection [203,243]. These studies expand our understanding to include how compounds are utilized to fuel the production of bioenergetic and biosynthetic intermediates during infection. Understanding how these alterations are directed by each virus will help us identify new targets for therapeutic development and possibly reveal common targets for broad antivirals.

In conclusion, an integrative approach involving genomics, transcriptomics, proteomics, and metabolomics will not only strengthen our understanding of the pathogenesis of these GHVs but also mark significant progress in the development of targeted diagnostics and therapeutics. The homology of MHV-68 with both EBV and KSHV provides the opportunity to derive insights from in vivo models, potentially enhancing our understanding of GHV infection dynamics. These advancements will support future research initiatives that address the complexities of gammaherpesvirus infections and their associated diseases while supporting the discovery of promising drug targets.

Despite the significant advancement of next-generation ‘omics’ technologies, many questions remain unresolved. The spatial and temporal coordination of gene and protein expression during infection, the influence of host genetic background on viral pathogenesis, the role of tissue-specific metabolic environments, and the long-term consequences of persistent infection on host cellular physiology are all active areas of investigation. Additionally, the growing field of single-cell and spatial omics promises to refine our understanding of viral heterogeneity, host cell tropism, and the microenvironmental context of infection.

## Figures and Tables

**Table 1:** Summarizing omics techniques used to understand herpesvirus biology during infection.

	Omics Technique	Virus	References
Genomics	Sanger sequencing	EBV	[71,72]
		MHV-68	[2]
	Cycle sequencing	MHV-68	[61]
	Shotgun sequencing	KSHV	[16,74,75]
	Dideoxynucleotide/M13 sequencing	EBV	[25]
	Sanger whole-genome sequencing	EBV	[76,81]
		KSHV	[83]
	High-throughput sequencing	EBV	[77]
	Single nucleotide polymorphism analysis	EBV	[79]
	Deep sequencing	EBV	[80]
	Genome-wide association study (GWAS)	EBV	[87]
	Next-Generation sequencing (NGS)	EBV	[88]
		KSHV	[83,91]
	Ion Torrent sequencing	KSHV	[92]
	Assay for Transposase-Accessible Chromatin sequencing (ATAC-seq)	EBV	[103,104,105]
	Chromatin immunoprecipitation sequencing (ChIP-seq)	EBV	[5,99,103,105,108,112]
		KSHV	[116]
		MHV68	[101,121]
	Reduced Representation Bisulfite Sequencing (RRBS)	EBV	[105,106]
	Whole-genome bisulfite sequencing	EBV	[104,109,246,247]
	Whole-exome sequencing (WES)	EBV	[82,104]
	Methylation capture sequencing	EBV	[110]
	Infinium MethylationEPIC BeadChip	EBV	[111]
	ChIP-on-chip analysis	KSHV	[96,113,114,115]
	MAPit single-molecule footprinting and bisulfite sequencing	KSHV	[117]
	CUT&RUN and GRO-seq	KSHV	[63,118,119]
	Capture Hi-C	KSHV	[63,248]
Transcriptomics	Bulk RNA sequencing	EBV	[108,130,131,133,135,136,141,143,165]
		KSHV	[17,119,137,138,145,146,147,167,168,249,250]
		MHV-68	[42,43,132,148]
	CUT&RUN sequencing	KSHV	[118]
	DNA microarrays	KSHV	[251,252]
		MHV-68	[139]
	Global cDNA array	MHV-68	[41]
	454 sequencing	MHV-68	[140]
	Single-cell RNA sequencing (scRNA-seq)	EBV	[149,150,151,152,153,162,163]
		KSHV	[154,155,156,157,158]
	Spatial transcriptomics	EBV	[162,163]
		KSHV	[164]
	Cap Analysis of Gene Expression sequencing (CAGE-seq)	EBV	[142]
		KSHV	[167,168]
	Deep CAGE	EBV	[165]
		MHV-68	[132]
	Polyadenylation sequencing (pA-seq)	EBV	[142,165]
		KSHV	[170]
		MHV-68	[132]
	Long-Read Sequencing (LRS)	EBV	[142]
		KSHV	[168]
		MHV-68	[132]
	Single-Molecule Real-Time Sequencing (SMRT)	KSHV	[167]
	RNA Annotation and Mapping of Promoters for the Analysis of Gene Expression (RAMPAGE)	KSHV	[166,168]
	Thiol(SH)-linked alkylation for the metabolic sequencing of RNA (SLAM-seq)	KSHV	[119]
Proteomics	Liquid Chromatography-Mass Spectrometry (LC-MS)	EBV	[189,226]
		KSHV	[220]
	MS/MS	EBV	[208]
	LC-MS/MS	EBV	[31,195,201,211]
		KSHV	[38,190,202,203,209]
		MHV-68	[44]
	1D gel/nano LC-MS/MS	MHV-68	[192]
	nano-LC-MS/MS	EBV	[18]
		MHV-68	[173]
	HPLC-MS	KSHV	[220]
	UHPLC-MS	EBV	[182,198]
	UPLC-MS/MS	EBV	[180]
	UHPLC-UHR-QqTOF LC/MS	KSHV	[181]
	tandem micro-LC/MS-MS	MHV-68	[193]
	HR-ESI-MS/MS	EBV	[225]
	MALDI-MS	KSHV	[37]
	MALDI-TOF MS	EBV	[182]
		KSHV	[214,215]
		MHV-68	[193]
	AP-MS	KSHV	[22]
	IP-MS	KSHV	[223]
	Rapid immunoprecipitation mass spectrometry of endogenous protein (RIME)	KSHV	[119,224]
	Proteomics of Isolated Chromatin fragments (PICh) MS	KSHV	[191]
	Yeast two-hybrid assay (Y2H)	EBV	[218]
		KSHV	[217]
		MHV-68	[19]
	Protein microarray	EBV	[184]
	Data-independent acquisition (DIA-MS)	EBV	[226]
	SILAC LC-MS/MS	EBV	[179,194,200,221]
		KSHV	[196,216]
	Gel-enhanced LC-MS (GeLC-MS/MS)	EBV	[221]
	TMT LC-MS/MS	KSHV	[171]
		KSHV	[171]
	TMT-nano-UHPLC-MS/MS	KSHV	[222]
	iTRAQ MS/MS	EBV	[174]
	iTRAQ-coupled HPLC-MS/MS	KSHV	[172]
	iTRAQ-2D LC-MS/MS	EBV	[186]
	HPLC-coupled high-resolution MS/MS	MHV-68	[173]
Metabolomics	LC-MS/MS	EBV	[20,103,238,239]
		KSHV	[203]
	LC-MS	EBV	[108,243]
		KSHV	[241,242]
		MHV-68	[45]
	GC-MS	EBV	[238,244]
	HPLC	EBV	[244]
	UHPLC-MS	EBV	[245]
		KSHV	[240,245]
	Nuclear magnetic resonance (NMR)	EBV	[21,253]

## Data Availability

No new data were created or analyzed in this study. Data sharing is not applicable to this article.

## Abbreviations

Affinity purification-mass spectrometry (AP-MS); Antigen F (HLA-F) adjacent transcript 10 (FAT10); Assay for Transposase-Accessible Chromatin sequencing (ATAC-seq); Burkitt’s lymphoma (BL); Cap Analysis of Gene Expression Sequencing (CAGE-seq); Carbamoyl-phosphate synthetase 2, aspartate transcarbamoylase, and dihydroorotase (CAD); Chromatin immunoprecipitation sequencing (ChIP-seq); Co-affinity purification (Co-AP); Deubiquitinating enzyme (DUB); DNA damage response (DDR); Epstein–Barr virus (EBV); EBV-associated GC (EBVaGC); Gammaherpesviruses (GHVs); Gas Chromatography-Mass Spectrometry (GCMS); Gastric cancer (GC); Genome-wide association study (GWAS); Heat shock protein (HSP); Hemophagocytic lymphohistiocytosis (HLH); Herpesvirus saimiri (HVS); High-performance LC (HPLC); High-throughput sequencing; Hodgkin’s lymphoma (HL); HSP70–HSP90 organizing protein (Hop); Human cytomegalovirus (HCMV); Human herpesvirus-8 (HHV-8); Immunoprecipitation (IP); Infectious mononucleosis (IM); Ion-based proteome-integrated solubility alteration (I-PISA); Isobaric tags for relative and absolute quantitation (iTRAQ); Kaposi’s Sarcoma (KS); Kaposi’s Sarcoma Inflammatory Cytokine Syndrome (KICS); Kaposi’s sarcoma-associated herpesvirus (KSHV); Laser Capture Microdissection (LCM); Latency-Associated Nuclear Antigen (LANA); Liquid chromatography-mass spectrometry (LC-MS); Liquid chromatography-tandem mass spectrometry (LC-MS/MS); Long-read sequencing (LRS); Lymphoblastoid cell line (LCL); Mass spectrometry (MS); Matrix metalloproteinases (MMPs); Matrix-assisted laser desorption ionization–time of flight mass spectrometry (MALDI-TOF); Mitogen-Activated Protein Kinase (MAPK) pathway; Multicentric Castleman Disease (MCD); Murine gammaherpesvirus 68 (MHV-68); Nasopharyngeal carcinoma (NPC); Next-generation sequencing (NGS); Normal oral keratinocytes (NOKs); Nuclear magnetic resonance (NMR) spectroscopy; Open reading frames (ORFs); PD-1/PD-L1 axis (programmed cell death protein-1 and its ligand); Peripheral Blood Mononuclear Cells (PBMC); Polyadenylation sequencing (pA-seq); Post-translational modifications (PTM); Primary Effusion Lymphoma (PEL); Protein–protein interactions (PPI); Reduced Representation Bisulfite Sequencing (RRBS); Replication and transcription activator (RTA); RNA sequencing (RNA-seq); Single-cell RNA sequencing (scRNA-sequencing) (Transcriptomics); single-cell sequencing (sc-Seq) (Genomics); Single-molecule real-time sequencing (SMRT); Small ubiquitin-related modifier (SUMO); Spatial transcriptomics (ST); Stable Isotope Labeling by Amino Acids in Cell Culture (SILAC); SUMO-interacting motif (SIM); Tandem Mass Tag (TMT); Tandem MS (or MS/MS); Thermal proximity coaggregation (TPCA)–MS; Thiol(SH)-linked alkylation for the metabolic sequencing of RNA (SLAM-seq); Tumor microenvironment (TME); Ubiquitin-fold modifier 1 (UFM); Ultra-high performance LC (UHPLC); Whole-exome sequencing (WES); Wild-type (WT) KSHV; Yeast two-hybrid assay (Y2H).
